# Assessment of the Prevalence of Hypertension and Its Associated Risk Factors in a Rural Community in Gwalior District, India

**DOI:** 10.7759/cureus.113238

**Published:** 2026-07-23

**Authors:** Bishwajeet Thakur, Kirti Shrivastava, Priyanka Diwan, Amit Agrawal, Avadhesh Diwakar, Jitendra Verma

**Affiliations:** 1 Community Medicine, Atal Bihari Vajpayee Government Medical College, Vidisha, Vidisha, IND; 2 Community Medicine, Gajra Raja Medical College, Gwalior, Gwalior, IND; 3 Anesthesia, District Hospital Niwari, Datia, IND

**Keywords:** hypertension, non-communicable diseases (ncds), prevalence, risk factors, rural population

## Abstract

Background: Hypertension is a major non-communicable disease and a leading contributor to cardiovascular morbidity and mortality. Its burden is increasing in rural India due to rapid lifestyle and epidemiological transitions.

Objectives: This study aims to assess the prevalence of hypertension and its associated risk factors among adults in a rural community in Gwalior district.

Methods: A community-based cross-sectional study was conducted from May 2023 to April 2024 among 360 adults selected through multistage sampling. Data were collected using a semi-structured questionnaire covering sociodemographic, dietary, and behavioral factors. Anthropometric measurements and blood pressure were recorded using standard procedures. Hypertension was defined as systolic blood pressure (SBP) ≥ 140 mmHg and/or diastolic blood pressure (DBP) ≥ 90 mmHg or current antihypertensive treatment. Data were analyzed using descriptive statistics and the chi-square test.

Results: Among 360 participants, 152 (42.2%) were aged 36-50 years and 180 (50.0%) were male. Hypertension was present in 139 (38.6%) participants, including stage 1 hypertension in 100 (27.8%) and stage 2 hypertension in 39 (10.8%). Pre-hypertension was observed in 106 (29.4%), while 115 (31.9%) had normal blood pressure. Overweight and obesity were found in 119 (33.1%) and 65 (18.1%) participants, respectively. Increased waist circumference and waist-to-hip ratio (WHR) were observed in 162 (45.0%) and 157 (43.6%) participants, respectively. Extra salt intake was reported by 212 (58.9%), smoking by 99 (27.5%), alcohol consumption by 106 (29.4%), and smokeless tobacco use by 120 (33.3%) participants. Significant associations were found between hypertension and male sex, overweight/obesity, excess salt intake, smoking, alcohol consumption, and family history of hypertension (p < 0.05).

Conclusion: Hypertension and pre-hypertension affected over two-thirds of the study population. Targeted screening, lifestyle modification, dietary interventions, and strengthened primary healthcare services are needed for early detection and prevention of hypertension.

## Introduction

Hypertension is one of the leading non-communicable diseases worldwide and remains a major contributor to cardiovascular morbidity and mortality. Because it often progresses without noticeable symptoms until complications develop, it is commonly referred to as the "silent killer." Uncontrolled hypertension substantially increases the risk of stroke, myocardial infarction, heart failure, and chronic kidney disease. The rising prevalence of hypertension has been driven by population aging, rapid urbanization, demographic transition, and widespread lifestyle changes, particularly in low- and middle-income countries such as India [1]. Owing to its high disease burden and preventable nature, effective prevention and control of hypertension are important public health priorities.

India has witnessed a steady increase in the prevalence of hypertension over recent decades. National surveys estimate that approximately one in four adults is affected, although prevalence varies by age, geographic location, and socioeconomic status [2]. Among individuals aged 15-49 years, hypertension affects about 11.3% of the population, with a slightly lower prevalence reported in rural areas (10.6%) [3]. In contrast, the prevalence increases markedly to approximately 41.9% among adults aged 45 years and older [4]. Furthermore, a systematic review estimated a pooled prevalence of 27.6% in rural India, indicating that hypertension is no longer confined to urban populations and has become an important public health concern across diverse settings [5].

Hypertension prevalence varies considerably across regions of India. A community-based study in rural Bihar found a prevalence of 24.55%, with another 25.35% classified as pre-hypertensive [6]. Similarly, studies in rural Maharashtra and rural Delhi reported rates of 29.99% and 14.1%, respectively [7,8]. These regional differences likely reflect variations in population characteristics, diet, lifestyle, socioeconomic conditions, and healthcare access. This highlights the need for district-level epidemiological studies to provide locally relevant evidence for planning effective prevention and control strategies.

Hypertension develops from a complex interaction of non-modifiable and modifiable risk factors. Advancing age, sex, and hereditary predisposition are key non-modifiable determinants. Modifiable risk factors include excess dietary salt, overweight and obesity, physical inactivity, tobacco use, alcohol consumption, and metabolic abnormalities. Studies consistently identify increasing age, higher body mass index (BMI), tobacco and alcohol use, and excessive salt intake as significant predictors of hypertension in rural populations [3,6,9]. Additionally, educational attainment, occupation, and socioeconomic status may affect both exposure to these risk factors and access to healthcare services [2].

Many individuals with hypertension in India remain undiagnosed, untreated, or inadequately controlled, especially in rural areas. Studies report low awareness, treatment, and blood pressure control among socioeconomically disadvantaged groups [4,10]. Delayed diagnosis often results from poor health literacy, limited screening, restricted healthcare access, and poor treatment adherence [10]. As a result, many continue to suffer preventable cardiovascular complications despite effective interventions.

Although several national and regional studies have described the epidemiology of hypertension, district-specific data from central India remain limited. Gwalior district is undergoing demographic and lifestyle transitions that may influence both the prevalence of hypertension and its associated risk factors. Reliable local evidence is essential for designing targeted interventions, strengthening primary healthcare services, and supporting the implementation of community-based non-communicable disease control programs. However, published data on the prevalence of hypertension and its associated risk factors among adults residing in the rural areas of Gwalior district are scarce.

Therefore, the present study was undertaken to estimate the prevalence of hypertension and assess its association with selected sociodemographic, anthropometric, behavioral, dietary, and family history-related factors among adults residing in rural Gwalior. The findings are expected to provide district-level evidence to support early detection, lifestyle modification, and the planning of effective community-based hypertension prevention and control strategies.

## Materials and methods

Study design and setting

A community-based cross-sectional study was conducted to assess the prevalence of hypertension and its associated risk factors among adults residing in rural areas of Gwalior district, Madhya Pradesh. The study was carried out in the rural field practice area attached to the Department of Community Medicine, Gajra Raja Medical College, Gwalior. Data were collected over a one-year period from May 1, 2023, to April 30, 2024, among permanent residents aged 18 years and above living in the selected rural communities of the district.

Sample size calculation

The sample size was calculated using the Cochran formula for prevalence studies:

$n=\frac{4pq}{L^{2}}$

where n is the required sample size, p is the estimated prevalence (%), q = 100 - p, and L is the allowable error (absolute precision). Based on findings from a previous study conducted in a rural population [11], the prevalence of hypertension was assumed to be 25%; therefore, p = 25 and q = 75. The allowable error was taken as 20% of the estimated prevalence, corresponding to an absolute precision of 5%. The minimum calculated sample size was 300 participants. After accounting for a 20% non-response rate, the final sample size was increased to 360 participants.

Sampling technique

A multistage sampling technique was employed to select the study participants. In the first stage, Gwalior district, comprising four administrative blocks (Morar, Ghatigaon, Dabra, and Bhitarwar), was considered as the sampling frame. All four blocks were included in the study to ensure representation of the entire rural field practice area.

In the second stage, one village from each administrative block was selected using simple random sampling by the lottery method. Accordingly, Hastinapur, Barai, Jourasi, and Bela villages were selected from Morar, Ghatigaon, Dabra, and Bhitarwar blocks, respectively.

In the third stage, a list of households in each selected village was prepared with the assistance of local health workers. Forty-five households from each village were selected by systematic random sampling. Within each selected household, two eligible adults (preferably the head of the household and the spouse) aged 18 years or older were included. If more than two eligible adults were present, the participants were selected by simple random sampling. If an eligible individual was unavailable after two visits or declined participation, the next eligible household was selected to maintain the required sample size.

**Figure 1:**
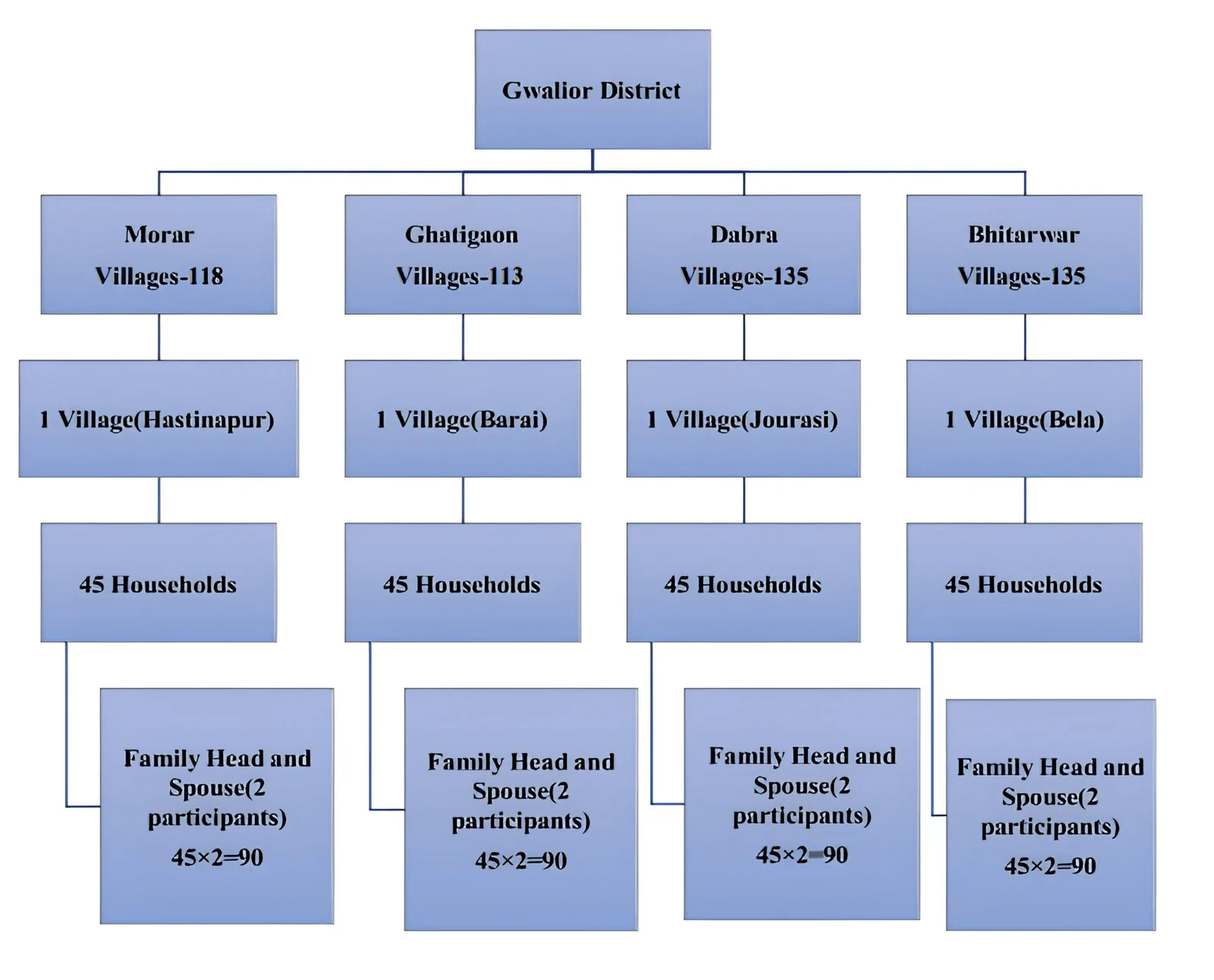
Multistage sampling technique used for the selection of study participants in rural Gwalior district, Madhya Pradesh The figure illustrates the multistage sampling procedure adopted for the study. Gwalior district was stratified into four blocks (Morar, Ghatigaon, Dabra, and Bhitarwar). One village was selected from each block (Hastinapur, Barai, Jourasi, and Bela, respectively). Forty-five households were selected by systematic random sampling from each village, and two eligible adults were selected from each household according to the study protocol, yielding 90 participants from each village and a total sample of 360 participants.

This sampling procedure resulted in the enrolment of 90 participants from each village, giving a total sample of 360 participants (Figure 1).

As all four administrative blocks of the study area were included and probability-based sampling methods were used at each stage, the risk of selection bias was minimized. However, the preferential inclusion of household heads and spouses may have introduced some selection bias, which has been acknowledged as a limitation of the study. Although multistage sampling was adopted, a design effect was not applied because the sampling units were selected from geographically distinct villages with similar population characteristics, and the study was designed as a descriptive community-based prevalence survey rather than a complex population survey.

Inclusion and exclusion criteria

Permanent residents of the selected rural areas of Gwalior district aged 18 years and above were eligible for inclusion in the study. Individuals who were seriously ill, unable to participate in the interview, or unavailable after two household visits were excluded from the study.

Data collection procedure

Data were collected from May 2023 to April 2024 using a pre-designed, semi-structured, interviewer-administered questionnaire (Appendices). The questionnaire was pre-tested in a rural population outside the study area to assess its clarity, feasibility, and completeness, and minor modifications were made before the final survey. It included information on sociodemographic characteristics, dietary habits, physical activity, tobacco and alcohol use, family history of hypertension, comorbid conditions, and anthropometric measurements.

Face-to-face interviews were conducted during household visits by the principal investigator only using paper-based questionnaires. Written informed consent was obtained from each participant before the interview. Blood pressure and anthropometric measurements were recorded according to standardized procedures, and completed questionnaires were checked daily for completeness and consistency to ensure data quality.

Anthropometric and blood pressure measurements

The sphygmomanometer and weighing scale were checked and calibrated periodically throughout the study according to standard procedures. Anthropometric measurements were taken following standard procedures. Height and weight were measured to calculate BMI. Waist circumference and waist-to-hip ratio (WHR) were also measured to assess central obesity.

Blood pressure was measured with a standard sphygmomanometer. Participants were seated comfortably and rested for at least five minutes before blood pressure measurement. Two readings were obtained at five-minute intervals, and the average value was used for analysis.

Blood pressure levels were classified according to the Joint National Committee (JNC) guidelines [12]. Individuals with systolic blood pressure (SBP) less than 120 mmHg and diastolic blood pressure (DBP) less than 80 mmHg were classified as normotensive. Pre-hypertension was defined as systolic blood pressure of 120-139 mmHg or diastolic blood pressure of 80-89 mmHg. Stage 1 hypertension was defined as systolic blood pressure of 140-159 mmHg or diastolic blood pressure of 90-99 mmHg, while stage 2 hypertension was defined as systolic blood pressure of 160 mmHg or higher or diastolic blood pressure of 100 mmHg or higher.

Operational definitions

Hypertension was defined as a systolic blood pressure of 140 mmHg or higher and/or a diastolic blood pressure of 90 mmHg or higher, or in previously diagnosed individuals receiving antihypertensive treatment [12]. Overweight and obesity were classified according to body mass index criteria using standard recommendations [13].

Statistical analysis

The collected data were entered into Microsoft Excel 2019 (Microsoft Corp., Redmond, WA) and analyzed using Jamovi version 2.6.44 (The Jamovi Project, Sydney, Australia). Descriptive statistics, including frequencies and percentages, were used to summarize the study findings. Associations between hypertension and selected risk factors were assessed using the chi-square test. A p-value of less than 0.05 was considered statistically significant.

Ethical considerations

The study was conducted in accordance with the ethical principles outlined in the Declaration of Helsinki, including respect for participants' autonomy, voluntary participation, informed consent, confidentiality, privacy, and the right to withdraw from the study at any stage without any adverse consequences. Ethical approval was obtained from the Institutional Ethics Committee of Gajra Raja Medical College, Gwalior, prior to the commencement of the study (approval number: 93/IEC-GRMC/2023).

Confidentiality and privacy of participant information were strictly maintained throughout the study. Personal identifiers were removed from the analytical dataset, and all study records were stored securely with access restricted to the research team. Interviews and blood pressure measurements were conducted in a private setting to ensure participant comfort, dignity, and confidentiality.

Handling of missing data

Data collection forms were reviewed daily to ensure completeness and consistency. Missing or unclear responses identified during field visits were verified immediately when feasible. Participants lacking information on key study variables, such as blood pressure measurements and major risk factor assessments, were excluded from the final analysis. The proportion of missing data was minimal and did not materially influence the study findings. Consequently, statistical imputation techniques were not applied, and analyses were conducted using only complete-case data.

## Results

A total of 360 participants were included in the study. Participants were distributed across four age groups: 29 (8.1%) were aged 18-35 years, 152 (42.2%) were aged 36-50 years, 141 (39.2%) were aged 51-65 years, and 38 (10.6%) were aged 66-80 years. Male and female participants were equally represented (180, 50.0% each). Regarding educational status, 207 (57.5%) participants were literate, whereas 153 (42.5%) were illiterate. With respect to socioeconomic status, 136 (37.8%) participants belonged to the lower middle class, followed by the middle class (120, 33.3%), upper middle class (66, 18.3%), lower class (20, 5.6%), and upper class (18, 5.0%). The sociodemographic characteristics of the study participants are presented in Table 1.

**Table 1:** Sociodemographic characteristics of the study participants (N = 360) Distribution of study participants according to age, sex, educational status, and socioeconomic status. Values are expressed as number (n) and percentage (%) of the total study population (N = 360). Socioeconomic status was assessed using the Modified Kuppuswamy Socioeconomic Scale.

Variable	Category	n	%
Age group (years)	18-35	29	8.1
	36-50	152	42.2
	51-65	141	39.2
	66-80	38	10.6
Sex	Male	180	50.0
	Female	180	50.0
Education	Illiterate	153	42.5
	Literate	207	57.5
Socioeconomic status	Upper	18	5.0
	Upper middle	66	18.3
	Middle	120	33.3
	Lower middle	136	37.8
	Lower	20	5.6

Regarding anthropometric characteristics, 24 (6.6%) participants were underweight, 152 (42.2%) had normal body mass index (BMI), 119 (33.1%) were overweight, and 65 (18.1%) were obese. Increased waist circumference and waist-to-hip ratio were observed in 162 (45.0%) and 157 (43.6%) participants, respectively. The anthropometric characteristics of the study participants are presented in Table 2.

**Table 2:** Distribution of study participants according to anthropometric characteristics (N = 360) Data are presented as frequency (n) and percentage (%).BMI was categorized as underweight, normal, overweight, and obese according to standard World Health Organization criteria. Waist circumference and waist-hip ratio were classified as normal or increased based on recommended cut-off values for central obesity. Anthropometric measurements were obtained using standardized procedures. BMI: body mass index

Variable	Category	n	%
Body mass index	Underweight	24	6.6
	Normal	152	42.2
	Overweight	119	33.1
	Obese	65	18.1
Waist circumference	Normal	198	55.0
	Increased	162	45.0
Waist-to-hip ratio	Normal	203	56.4
	Increased	157	43.6

Blood pressure assessment showed that 115 (31.9%) participants had normal blood pressure, while 106 (29.4%) were prehypertensive. Stage 1 and stage 2 hypertension were observed in 100 (27.8%) and 39 (10.8%) participants, respectively. Overall, 139 (38.6%) participants had hypertension. The distribution of participants by blood pressure category is presented in Table 3.

**Table 3:** Distribution of study participants according to blood pressure categories (N = 360) Values are expressed as number (n) and percentage (%) of the total study population. Blood pressure categories were classified according to standard hypertension guidelines as normal, pre-hypertension, stage 1 hypertension, and stage 2 hypertension. The table shows the prevalence of different blood pressure categories among the study participants at the time of assessment.

Blood pressure category	n	%
Normal	115	31.9
Pre-hypertension	106	29.4
Stage 1 hypertension	100	27.8
Stage 2 hypertension	39	10.8

A vegetarian diet was reported by 217 (60.3%) participants, while 143 (39.7%) consumed a non-vegetarian diet. Junk food consumption was reported by 229 (63.6%) participants, and 212 (58.9%) reported additional salt intake. Adequate sleep and regular physical activity were reported by 268 (74.4%) and 188 (52.2%) participants, respectively. Smoking, alcohol consumption, and smokeless tobacco use were reported by 99 (27.5%), 106 (29.4%), and 120 (33.3%) participants, respectively. A family history of hypertension was present in 47 (13.1%) participants, while 204 (56.7%) had one or more comorbid conditions. The distribution of lifestyle and clinical risk factors among the study participants is presented in Table 4.

**Table 4:** Distribution of study participants according to lifestyle and clinical risk factors for hypertension (N = 360) The table summarizes the distribution of dietary habits, sleep adequacy, physical activity, substance use (smoking, alcohol consumption, and smokeless tobacco use), family history of hypertension, and presence of comorbidities among study participants. These variables were assessed as potential risk factors associated with hypertension in the study population. Values are expressed as number (n) and percentage (%) of the total study population (N = 360).

Variable	Category	n	%
Diet type	Vegetarian	217	60.3
	Non-vegetarian	143	39.7
Junk food consumption	Yes	229	63.6
	No	131	36.4
Extra salt intake	Yes	212	58.9
	No	148	41.1
Adequate sleep	Yes	268	74.4
	No	92	25.6
Physical activity	Yes	188	52.2
	No	172	47.8
Smoking	Yes	99	27.5
	No	261	72.5
Alcohol consumption	Yes	106	29.4
	No	254	70.6
Smokeless tobacco use	Yes	120	33.3
	No	240	66.7
Family history of hypertension	Present	47	13.1
	Absent	313	86.9
Presence of any comorbidity	Yes	204	56.7
	No	156	43.3

Among participants with comorbidities, diabetes mellitus was reported in 56 (27.5%), followed by cardiovascular disease in 48 (23.5%), chronic obstructive pulmonary disease in 46 (22.5%), kidney disease in 44 (21.6%), cerebrovascular disease in 34 (16.7%), arthritis in 33 (16.2%), asthma in 26 (12.7%), and peripheral vascular disease in 14 (6.9%) participants. The distribution of comorbid conditions among participants is presented in Table 5.

**Table 5:** Distribution of comorbid conditions among participants with comorbidities (n = 204) Data are presented as frequency (n) and percentage (%). The table summarizes the prevalence of various self-reported or documented comorbid conditions among participants with at least one comorbidity (n = 204). Percentages were calculated using the total number of participants with comorbidities as the denominator. Multiple comorbid conditions may have been present in the same individual; therefore, percentages may not sum to 100%. COPD: chronic obstructive pulmonary disease

Comorbidity	n	%
Diabetes mellitus	56	27.5
Cardiovascular disease	48	23.5
COPD	46	22.5
Kidney disease	44	21.6
Cerebrovascular disease	34	16.7
Arthritis	33	16.2
Asthma	26	12.7
Peripheral vascular disease	14	6.9

Significant associations were observed between blood pressure classification and sex (χ² = 8.77, p = 0.032), body mass index (BMI) (χ² = 73.7, p < 0.001), extra salt intake (χ² = 37.9, p < 0.001), smoking (χ² = 21.0, p < 0.001), alcohol consumption (χ² = 26.0, p < 0.001), and family history of hypertension (χ² = 41.3, p < 0.001). The associations between selected risk factors and blood pressure categories are presented in Table 6.

**Table 6:** Association between selected sociodemographic and lifestyle risk factors and blood pressure categories among study participants (N = 360) Data are presented as number (n) and percentage (%) of participants within each category. Associations between blood pressure categories (normal, pre-hypertension, stage 1 hypertension, and stage 2 hypertension) and selected risk factors were evaluated using the chi-square (χ²) test. Significant associations were observed for sex, BMI, extra salt intake, smoking, alcohol consumption, and family history of hypertension. Statistical significance was considered at p < 0.05. HTN: hypertension, BMI: body mass index, χ²: chi-square statistic *p < 0.05, ***p < 0.001

Variable	Category	Normal (n (%))	Pre-hypertension (n (%))	Stage 1 hypertension (n (%))	Stage 2 hypertension (n (%))	χ²	p-value
Sex	Male	49 (27.2)	48 (26.7)	59 (32.8)	24 (13.3)	8.77	0.032*
	Female	66 (36.7)	58 (32.2)	41 (22.8)	15 (8.3)		
Category of BMI	Underweight	9 (37.5)	8 (33.3)	6 (25.0)	1 (4.2)	73.7	<0.001***
	Normal	75 (49.3)	52 (34.2)	16 (10.5)	9 (5.9)		
	Overweight	19 (16.0)	31 (26.1)	54 (45.4)	15 (12.6)		
	Obese	12 (18.5)	15 (23.1)	24 (36.9)	14 (21.5)		
Extra salt intake	Yes	42 (19.5)	75 (34.9)	70 (32.6)	28 (13.0)	37.9	<0.001***
	No	73 (50.3)	31 (21.4)	30 (20.7)	11 (7.6)		
Smoking	Yes	20 (20.2)	23 (23.2)	37 (37.4)	19 (19.2)	21.0	<0.001***
	No	95 (36.4)	83 (31.8)	63 (24.1)	20 (7.7)		
Alcohol consumption	Yes	23 (17.2)	40 (29.9)	51 (38.1)	20 (14.9)	26.0	<0.001***
	No	92 (40.7)	66 (29.2)	49 (21.7)	19 (8.4)		
Family history of hypertension	Present	8 (17.0)	3 (6.4)	21 (44.7)	15 (31.9)	41.3	<0.001***
	Absent	107 (34.2)	103 (32.9)	79 (25.2)	24 (7.7)		

## Discussion

The present community-based study demonstrated that hypertension affected 38.6% of adults residing in rural Gwalior, while an additional 29.4% were classified as pre-hypertensive. Consequently, more than two-thirds of the study population either had hypertension or were at increased risk of developing it. These findings emphasize the considerable burden of hypertension in rural communities and reflect the ongoing epidemiological transition toward non-communicable diseases in India.

The prevalence observed in the present study is broadly consistent with findings from other regions of the country. The Puducherry STEPS survey reported a hypertension prevalence of 33.6% [14], whereas national estimates have ranged between 22.6% and 28.1% [15,16]. Higher prevalence has been documented among older adults, reaching 41.9% in individuals aged 45 years and above [4]. Conversely, some rural studies have reported lower prevalence estimates of approximately 21.4% [15]. The comparatively higher prevalence identified in the present study may reflect ongoing demographic and lifestyle changes, including increasing obesity, dietary modifications, and reduced physical activity. Similar upward trends in hypertension prevalence and systolic blood pressure have also been documented in longitudinal studies from rural North India [17].

Advancing age was significantly associated with higher blood pressure categories in the study population. Participants in older age groups exhibited greater proportions of stage 1 and stage 2 hypertension, consistent with previous evidence demonstrating a progressive increase in hypertension prevalence with advancing age. National data indicate that nearly half of adults aged 60 years or older are hypertensive [15], while comparable observations have also been reported among tribal populations [18]. These findings are consistent with the cumulative effects of vascular ageing and prolonged exposure to cardiovascular risk factors.

Male participants exhibited a greater burden of hypertension than female participants, with significantly higher proportions of stage 1 and stage 2 hypertension. Similar sex-related differences have been reported in several community-based studies and national surveys [15,19]. The higher prevalence among men may be associated with their greater exposure to behavioral risk factors, including tobacco use and alcohol consumption, both of which are recognized contributors to elevated blood pressure [10].

Measures of both generalized and central obesity showed a strong association with hypertension. More than half of the participants were either overweight or obese, while a substantial proportion had increased waist circumference and waist-to-hip ratio. These findings are consistent with national evidence demonstrating that excess body weight and abdominal adiposity are associated with hypertension [15]. The Longitudinal Ageing Study in India (LASI) also reported marked socioeconomic differences in central obesity [14]. Compared with earlier rural studies in which undernutrition predominated, the present findings suggest a transition toward obesity-related cardiovascular risk factors.

Dietary practices also appeared to be associated with blood pressure status. Nearly three-fifths of participants reported consuming additional dietary salt, which showed a significant association with hypertension. Excess sodium intake has consistently been identified as an important modifiable factor associated with hypertension and cardiovascular disease [15]. Furthermore, the high frequency of junk food consumption observed in this study reflects changing dietary patterns in rural communities, which may further contribute to the increasing burden of non-communicable diseases [14].

Behavioral risk factors were common in the study population. Approximately one-third of participants reported smoking, alcohol consumption, or smokeless tobacco use, and both smoking and alcohol intake were significantly associated with hypertension. These observations are consistent with evidence from South Asian populations demonstrating that unhealthy lifestyle behaviors are associated with increased cardiovascular risk [20]. The coexistence of multiple behavioral risk factors may further increase susceptibility to hypertension, particularly among vulnerable groups [10].

A positive family history was significantly associated with hypertension, suggesting that genetic susceptibility together with shared environmental and behavioral factors may influence blood pressure. Similar associations have been documented in national studies evaluating risk factors for hypertension [16].

The findings also highlight persistent challenges in hypertension control. A considerable proportion of individuals with hypertension remain unaware of their condition or do not receive adequate treatment, resulting in poor blood pressure control [21]. These challenges are particularly pronounced in rural settings, where barriers such as limited healthcare access, inadequate awareness, and poor treatment adherence continue to affect disease management [17]. Consequently, rural populations experience disproportionately higher cardiovascular morbidity and mortality than their urban counterparts [20].

Community-based interventions have demonstrated considerable potential for improving hypertension prevention and management. Evidence from an accredited social health activist (ASHA)-led program has shown meaningful improvements in blood pressure control among rural populations [22]. Strengthening such initiatives may improve hypertension prevention and management in rural communities.

Strengths

The present study has several strengths. It employed a community-based cross-sectional design with an adequate sample size and a multistage sampling technique. Standardized procedures were used for measuring blood pressure and anthropometric parameters. In addition, the study assessed a range of sociodemographic, behavioral, dietary, anthropometric, and family history-related factors associated with hypertension. The findings provide district-level data on the prevalence of hypertension and its associated factors among adults residing in rural Gwalior.

Limitations

The findings of this study should be interpreted in light of several limitations. First, the cross-sectional study design precludes establishing temporal or causal relationships between hypertension and the associated factors. Second, the study was conducted in selected rural areas of Gwalior district, which may limit the generalizability of the findings to other geographic regions or urban populations. Third, several lifestyle-related variables, including dietary salt intake, physical activity, smoking, alcohol consumption, and sleep patterns, were based on self-reported information and may therefore be subject to recall and social desirability bias. Fourth, blood pressure measurements were obtained during a single visit, making it possible that transient fluctuations or the white-coat effect influenced classification. Fifth, although multistage sampling was employed, selection bias cannot be completely excluded because participants were recruited from selected households. Sixth, only bivariate (chi-square) analysis was performed; therefore, potential confounding factors were not adjusted for, and independent associations could not be established. Finally, the absence of biochemical investigations and detailed dietary assessments limited a more comprehensive evaluation of metabolic and nutritional determinants of hypertension. Nevertheless, the study provides valuable district-level evidence regarding the burden of hypertension and its associated factors and offers useful information for planning targeted public health interventions in rural communities.

## Conclusions

The present study found a high prevalence of hypertension and pre-hypertension among adults residing in rural Gwalior, indicating a considerable burden of elevated blood pressure in the study population. Male sex, excess body weight, high dietary salt intake, tobacco smoking, alcohol consumption, and a family history of hypertension were significantly associated with hypertension. These findings highlight the importance of strengthening primary healthcare services through routine blood pressure screening, health education, promotion of healthy lifestyles, dietary modification, and appropriate follow-up of individuals with elevated blood pressure. Further longitudinal studies are warranted to better understand the temporal relationships between these factors and hypertension.

## Appendices

Table 7 presents the questionnaire used in the present study. 

**Table 7:** Study proforma used for data collection of sociodemographic, clinical, anthropometric, dietary, lifestyle, and family history variables The table presents the structured questionnaire and examination proforma used for data collection in the study. Information collected included sociodemographic characteristics, anthropometric measurements, blood pressure assessment, medical history, dietary practices, lifestyle factors, substance use, physical activity, comorbidities, and family history of hypertension. Blood pressure was measured twice using a standardized protocol, and the average of the two readings was used for classification. Socioeconomic status was assessed using the Modified B.G. Prasad Scale (October 2023).

S. No.	Variable	Response/category
1	Name of participant	
2	Age (years)	__________
3	Sex	Male / Female
4	Category	Unreserved (UR) / Other backward caste (OBC) / Scheduled caste (SC) / Schedule tribe (ST)
5	Address	__________
6	Religion	Hindu / Muslim / Christian / Sikh / Other
7	Educational status	Illiterate / Primary school / Middle school / High school / Higher secondary / Graduate and above
8	Occupation	Unemployed / Unskilled / Semi-skilled / Skilled / Clerk
9	Marital status	Married / Unmarried / Divorced / Widow(er) / Separated
10	Type of family	Single / Nuclear / Joint / Three-generation
11	Total members in family	__________
12	Total family income	__________
13	Per capita income	__________
14	Socioeconomic status (Modified B.G. Prasad Scale, October 2023)	Upper / Upper middle / Middle / Lower middle / Lower
15	General condition	Poor / Average / Good
16	Built	Thin / Average / Obese
17	Height (cm)	__________
18	Weight (kg)	__________
19	Body Mass Index (BMI)	Underweight (<18.5) / Normal (18.5-22.99) / Overweight (23-24.99) / Obese (≥25)
20	Waist circumference (cm)	__________
21	Hip circumference (cm)	__________
22	Waist-to-hip ratio (WHR)	__________
23	Pulse rate	__________
24	Temperature	__________
25	Blood pressure measurement	1st reading systolic blood pressure (SBP) ___ Diastolic blood pressure (DBP) ___ ; 2nd reading SBP ___ DBP ___ ; Average SBP ___ DBP ___
26	Previously diagnosed with hypertension	Yes / No
27	Place of treatment	Private doctor / Government hospital / District hospital / Primary health center (PHC) / Community health center (CHC)
28	Presence of disease other than hypertension	Yes / No
29	Type of comorbidity	Peripheral vascular disease / Kidney disease / Diabetes mellitus / Heart failure / Cerebrovascular disease / Myocardial infarction / Arthritis / COPD / asthma
30	Type of diet	Vegetarian / Non-vegetarian
31	Junk food consumption	Yes / No
32	Frequency of junk food consumption	____ days/week
33	Extra salt intake	Yes / No
34	Type of salt used	Iodized / Non-iodized
35	Type of cooking oil used	Mustard oil / Groundnut oil / Sunflower oil / Palm oil / Soybean oil / Ghee / Dalda / Other
36	Preferred food type	More spicy / Less spicy / Non-spicy / More spicy and more oily / Less spicy and less oily
37	Preventive dietary measures followed	Limit salt intake / Eat less spicy food / Avoid spicy and oily food / Eat less sugar / Avoid sweets / Eat more fruits and vegetables / Limit excess calories
38	Adequate sleep (6–8 h/night)	Yes / No
39	Reason for sleep deprivation	Stress / Anxiety / Poor sleeping environment / Shift work / Mental health condition
40	Alcohol consumption	Yes / No
41	Duration of alcohol consumption	<1 year / 1-2 years / 2-3 years / 3-5 years / ≥5 years
42	Frequency of alcohol consumption	____ days/month
43	Average alcohol intake	1-2 units / 2-3 units / 3-4 units / 4-6 units / ≥6 units
44	Smoking tobacco	Yes / No
45	Duration of smoking	<1 year / 1-2 years / 2-3 years / 3-5 years / ≥5 years
46	Number of cigarettes/bidis per day	__________
47	Smokeless tobacco consumption	Yes / No
48	Duration of smokeless tobacco use	<1 year / 1-2 years / 2-3 years / 3-5 years / ≥5 years
49	Type of smokeless tobacco	Tobacco pan masala / Tobacco with lime / Tobacco with pan / Betel quid / Khaini
50	Frequency of smokeless tobacco use	1-2 times/day / 2-3 times/day / 3-4 times/day / 4-5 times/day / ≥5 times/day
51	Physical activity	Yes / No
52	Type of physical activity	Brisk walking / Cycling / Housework / Running / Yoga
53	Duration of physical activity	30 minutes / 30-60 minutes / 60-90 minutes / 90-120 minutes / >120 minutes
54	Family history of hypertension	Yes / No
55	Side of family affected	Maternal / Paternal
56	Duration of hypertension in family member	1 year / 2-3 years / 3-4 years / 4-5 years / 5-10 years

## References

[1] Geldsetzer P, Manne-Goehler J, Theilmann M (2018). Diabetes and hypertension in India: a nationally representative study of 1.3 million adults. JAMA Intern Med.

[2] Moser KA, Agrawal S, Davey Smith G (2014). Socio-demographic inequalities in the prevalence, diagnosis and management of hypertension in India: analysis of nationally-representative survey data. PLoS One.

[3] Ghosh S, Kumar M (2019). Prevalence and associated risk factors of hypertension among persons aged 15-49 in India: a cross-sectional study. BMJ Open.

[4] Mohanty SK, Pedgaonkar SP, Upadhyay AK (2021). Awareness, treatment, and control of hypertension in adults aged 45 years and over and their spouses in India: a nationally representative cross-sectional study. PLoS Med.

[5] Anchala R, Kannuri NK, Pant H (2014). Hypertension in India: a systematic review and meta-analysis of prevalence, awareness, and control of hypertension. J Hypertens.

[6] Ranjan A, Pandey S, Kumar Nirala S (2025). Prevalence and factors associated with hypertension in rural areas of Naubatpur block of Patna District in Bihar, India: a population-based cluster cross-sectional study. BMC Public Health.

[7] Uthakalla VK, Naidana PS, Yendapu RS (2024). Prevalence of hypertension among the rural adult population in India: a systematic review and meta-analysis. Cureus.

[8] Kishore J, Gupta N, Kohli C (2016). Prevalence of hypertension and determination of its risk factors in rural Delhi. Int J Hypertens.

[9] Bhise MD, Patra S (2018). Prevalence and correlates of hypertension in Maharashtra, India: a multilevel analysis. PLoS One.

[10] Boro B, Banerjee S (2022). Decomposing the rural-urban gap in the prevalence of undiagnosed, untreated and under-treated hypertension among older adults in India. BMC Public Health.

[11] Kori S, Sahasrabuddhe AG, Arora VK (2021). Prevalence of hypertension and its risk factors among adults in rural community: a cross-sectional study. Natl J Community Med.

[12] Amsterdam EA, Venugopal S, Bui J (2016). Management of hypertension: JNC 8 and beyond. CVIA.

[13] (2026). Centers for Disease Control and Prevention: Adult BMI categories. https://www.cdc.gov/bmi/adult-calculator/bmi-categories.html.

[14] Sivanantham P, Sahoo J, Lakshminarayanan S (2021). Profile of risk factors for non-communicable diseases (NCDs) in a highly urbanized district of India: findings from Puducherry district-wide STEPS survey, 2019-20. PLoS One.

[15] Mohammad R, Bansod DW (2024). Hypertension in India: a gender-based study of prevalence and associated risk factors. BMC Public Health.

[16] Varghese JS, Venkateshmurthy NS, Sudharsanan N (2023). Hypertension diagnosis, treatment, and control in India. JAMA Netw Open.

[17] Kumar K, Misra S (2021). Sex differences in prevalence and risk factors of hypertension in India: evidence from the National Family Health Survey-4. PLoS One.

[18] Joseph P, Kutty VR, Mohan V (2022). Cardiovascular disease, mortality, and their associations with modifiable risk factors in a multi-national South Asia cohort: a PURE substudy. Eur Heart J.

[19] Shaikh R, Khan J (2021). Clustering of lifestyle risk factors among adult population in India: a cross-sectional analysis from 2005 to 2016. PLoS One.

[20] Riddell MA, Mini GK, Joshi R (2021). ASHA-led community-based groups to support control of hypertension in rural India are feasible and potentially scalable. Front Med (Lausanne).

[21] Babu BV, Hazarika CR, Raina SK (2024). Hypertension prevalence, awareness, treatment, control and risk factors in tribal population of India: a multi-centric cross-sectional study. J Racial Ethn Health Disparities.

[22] Ambade M, Kim R, Subramanian SV (2023). Socio-economic distribution of modifiable risk factors for cardiovascular diseases: an analysis of the national longitudinal ageing study in India. Prev Med.

